# Aging modulates amyloid clearance kinetics during anti-amyloid therapy: evidence from real-world serial amyloid PET

**DOI:** 10.3389/fnagi.2026.1801267

**Published:** 2026-05-08

**Authors:** Tomomichi Iizuka, Toshiaki Watanabe, Masashi Kameyama

**Affiliations:** 1 Center for Dementia, Fukujuji Hospital, Tokyo, Japan; 2 Department of Nuclear Medicine, Fukujuji Hospital, Tokyo, Japan; 3 AI and Theoretical Image Processing, Research Team for Neuroimaging, Tokyo Metropolitan Institute for Geriatrics and Gerontology, Tokyo, Japan

**Keywords:** aging, Alzheimer’s disease, amyloid PET, centiloid, lecanemab, perfusion SPECT, serial amyloid PET, treatment response

## Abstract

**Background:**

Anti-amyloid antibodies have been shown to reduce cerebral amyloid burden in early Alzheimer’s disease (AD), yet considerable interindividual variability in treatment-associated amyloid reduction has been observed. The biological factors underlying this variability remain unclear. In particular, the influence of aging on amyloid clearance dynamics during anti-amyloid therapy has not been well characterized in real-world clinical settings.

**Methods:**

We conducted a prospective observational study of 23 patients with early-stage AD receiving lecanemab who underwent serial ^18^F-flutemetamol amyloid PET at baseline and after 6 and 12 months. Amyloid burden was quantified in centiloid units. Cognitive outcomes were assessed using the Mini-Mental State Examination (MMSE) and Clinical Dementia Rating–Sum of Boxes (CDR-SB). Baseline cerebral perfusion was evaluated using ^123^I-IMP SPECT with three-dimensional stereotactic surface projection (3D-SSP) analysis.

**Results:**

All participants showed reductions in amyloid burden on serial PET; however, the magnitude of reduction varied substantially across individuals. Older patients tended to exhibit larger and more rapid reductions in amyloid burden, whereas younger patients demonstrated more modest decreases despite comparable baseline amyloid levels and standardized dosing. Amyloid reduction at 6 months strongly predicted the magnitude of reduction at 12 months, suggesting that early PET changes capture subsequent amyloid clearance trajectories. Cognitive decline occurred in a subset of patients despite substantial amyloid reduction and was associated with marked baseline temporo-parietal hypoperfusion on SPECT rather than insufficient amyloid removal.

**Conclusion:**

In this real-world cohort, aging appeared to influence the observable kinetics of amyloid reduction during anti-amyloid therapy. Early serial amyloid PET may provide useful information regarding longer-term amyloid dynamics, while baseline perfusion imaging may help identify patients with substantial downstream neurodegenerative burden who remain at risk for cognitive decline despite amyloid clearance. These findings highlight biological heterogeneity in treatment response and underscore the value of multimodal imaging for monitoring disease-modifying therapies in AD.

## 1 Introduction

Alzheimer’s disease (AD) is characterized by extracellular amyloid-β (Aβ) plaques and intracellular neurofibrillary tangles composed of hyperphosphorylated tau (Selkoe, 1991; Braak and Braak, 1991). According to the amyloid cascade hypothesis, aggregation of Aβ is considered an early initiating event that triggers downstream neurodegenerative processes (Hardy and Higgins, 1992). Among Aβ assemblies—monomers, oligomers, protofibrils, and fibrils—protofibrils represent a critical intermediate species bridging soluble oligomers and insoluble fibrils. Accumulating evidence indicates that soluble Aβ species, particularly oligomers and protofibrils, exert neurotoxicity through synaptic dysfunction, oxidative stress, and neuroinflammatory activation, and are closely linked to cognitive decline (Lannfelt et al., 2014; Ono et al., 2009; Chen et al., 2023).

Lecanemab is a humanized IgG1 monoclonal antibody that preferentially targets soluble Aβ protofibrils while showing lower affinity for plaque-associated Aβ (Van Dyck et al., 2023). Although protofibrils themselves cannot yet be routinely quantified in clinical practice, amyloid positron emission tomography (PET) provides a robust and widely available method for assessing treatment-associated changes in fibrillar amyloid burden (Nordberg, 2004; Villemagne et al., 2013; La Joie et al., 2019). Serial amyloid PET therefore offers an indirect but biologically informative window into amyloid dynamics during protofibril-targeting therapy.

As lecanemab is increasingly implemented in routine clinical practice, substantial interindividual variability has been observed in both amyloid reduction and cognitive trajectories. Notably, some patients exhibit pronounced amyloid clearance, whereas others show attenuated PET-detectable reductions, and cognitive decline may occur despite substantial amyloid removal. These observations raise fundamental questions regarding the biological factors that shape amyloid clearance dynamics during therapy, beyond differences in drug exposure or baseline amyloid burden.

Aging represents a plausible yet insufficiently characterized modifier of treatment-associated amyloid dynamics. Age-related differences in amyloid production, clearance capacity, blood–brain barrier function, and neurodegenerative burden may influence how amyloid-targeting interventions translate into measurable changes on longitudinal PET imaging. However, real-world data incorporating truly serial amyloid PET remain scarce, and the extent to which aging modulates amyloid clearance kinetics outside controlled trial settings is not well understood.

Therefore, in this prospective observational study, we investigated (i) whether the magnitude and kinetics of amyloid reduction during lecanemab therapy vary with age, (ii) whether early amyloid reduction predicts longer-term amyloid PET outcomes, and (iii) whether baseline cerebral perfusion deficits on single-photon emission computed tomography (SPECT) identify patients at risk of cognitive decline despite adequate amyloid reduction. To address these questions, we performed serial amyloid PET at baseline, 6 months, and 12 months, together with baseline perfusion SPECT and longitudinal cognitive assessment in a real-world clinical cohort.

## 2 Materials and methods

### 2.1 Study design and participants

This prospective observational study was conducted at the Center for Dementia, Fukujuji Hospital (Tokyo, Japan). Participants were consecutively enrolled between February 2024 and April 2025. Participants met the 2011 National Institute on Aging–Alzheimer’s Association (NIA-AA) criteria for probable Alzheimer’s disease (AD) dementia or mild cognitive impairment due to AD. Eligibility required a positive amyloid PET scan and early-stage disease severity defined as Clinical Dementia Rating (CDR) Global Score ≤ 1, CDR Sum of Boxes (CDR-SB) ≤ 8, and Mini-Mental State Examination (MMSE) score ≥ 22.

Exclusion criteria included neurological conditions associated with increased risk of amyloid-related imaging abnormalities (ARIA), such as transient ischemic attack, stroke, or seizure within the preceding 12 months, as well as other uncontrolled medical disorders that could affect safety or study outcomes. Patients with clinical features suggestive of other neurodegenerative disorders, including dementia with Lewy bodies, were excluded based on comprehensive clinical evaluation. In addition, participants with significant cerebrovascular lesions on brain MRI were excluded.

Tau biomarkers and APOE genotyping were not routinely available in this clinical cohort.

For exploratory analyses, participants were stratified into two groups according to age at treatment initiation ( < 70 years vs. ≥ 70 years). Baseline demographic and clinical characteristics were compared between age groups to evaluate potential confounding factors.

### 2.2 Treatment protocol

Lecanemab was administered intravenously at 10 mg/kg every 2 weeks according to routine clinical practice. Prior to treatment initiation, participants underwent a structured clinical interview including assessment of daily physical and social activities.

### 2.3 Amyloid PET acquisition and quantification

Amyloid PET was performed using ^18^F-flutemetamol. Scans were obtained at baseline and after 6 and 12 months of lecanemab therapy. PET data were processed using PMOD software (version 4.2), and global amyloid burden was quantified in centiloid units. Regional amyloid burden was also assessed using predefined cortical regions of interest. Change in amyloid burden over time was evaluated using within-subject comparisons.

### 2.4 Cognitive assessments

Global cognitive function and clinical severity were assessed longitudinally using the MMSE and CDR-SB at baseline and during follow-up visits.

To illustrate heterogeneity in clinical trajectories during the 6-month follow-up, patients were categorized into three groups (decline, stable, improvement) based on changes in CDR-SB and MMSE scores. Cognitive decline was defined as either an increase in CDR-SB ≥ 1.0 point or a decrease in MMSE ≥ 2 points between baseline and the 6-month follow-up. Cognitive improvement was defined as either a decrease in CDR-SB ≥ 1.0 point or an increase in MMSE ≥ 2 points. Patients not meeting either criterion were categorized as cognitively stable. These thresholds were chosen to reflect clinically meaningful cognitive changes reported in previous studies (Andrews et al., 2019; Han et al., 2000).

Cognitive assessments were conducted as part of routine clinical evaluations, and raters were not formally blinded to PET results.

### 2.5 Perfusion SPECT and 3D-SSP analysis

Baseline cerebral perfusion was assessed using ^123^I-IMP single-photon emission computed tomography (SPECT). Voxel-wise perfusion patterns were evaluated using three-dimensional stereotactic surface projection (3D-SSP) analysis to explore associations between baseline perfusion abnormalities and subsequent cognitive outcomes during therapy.

### 2.6 MRI monitoring for ARIA

Brain MRI was performed at baseline and serially during treatment to monitor for ARIA. Imaging included FLAIR sequences for ARIA-E and susceptibility-weighted imaging (SWI) for ARIA-H. All MRI scans were reviewed by two board-certified neuroradiologists, and discrepancies were resolved by consensus.

### 2.7 Outcome measures

The primary outcome was the change in global amyloid burden (centiloid units) from baseline to 6 and 12 months. Secondary outcomes included (i) regional amyloid changes, (ii) longitudinal changes in MMSE and CDR-SB, and (iii) associations between baseline 3D-SSP perfusion patterns and cognitive trajectory. We also examined whether early amyloid reduction at 6 months predicted longer-term reduction at 12 months.

### 2.8 Statistical analysis

Statistical analyses were conducted using Excel Statistics (Social Survey Research Information Co., Ltd., Tokyo, Japan).

For exploratory analyses, participants were stratified into younger and older groups based on age (<70 vs. ≥ 70). Baseline demographic and clinical characteristics were compared between the age groups to evaluate potential confounding factors using independent-samples *t*-tests for continuous variables and Fisher’s exact tests for categorical variables.

Paired t-tests were used for within-subject comparisons across time points. Pearson correlation coefficients were calculated to assess associations between continuous variables. Regional differences in amyloid reduction were examined using repeated-measures ANOVA with Bonferroni correction for multiple comparisons.

Given multiple comparisons, statistical significance was set at a two-sided p value < 0.01.

### 2.9 Ethics

The study was approved by the Ethical Review Board of Fukujuji Hospital (Approval No. 24027) and conducted in accordance with the Declaration of Helsinki. Written informed consent was obtained from all participants and/or their legally authorized representatives.

## 3 Results

### 3.1 Study population and baseline characteristics

A total of 25 patients were screened; after applying exclusion criteria, 23 participants were included in the final analysis. Of these 23 participants, 20 had amnestic mild cognitive impairment (MCI) and 3 had mild dementia. All 23 patients completed the 6-month follow-up, and 20 completed 12 months of treatment. Clinical characteristics and longitudinal cognitive outcomes are summarized in Table 1.

**TABLE 1:** Clinical characteristics and longitudinal cognitive outcomes.

	Baseline	6 months	12 months
Participants (n)	23	23	20
Age (y)	71.9 (7.0)	–	–
Gender (M/F)	5/18	5/18	4/16
MMSE	25.7 (1.4)	27.3 (2.6) ^†^	27.0 (2.9)
CDR-GS	0.54 (0.14)	0.59 (0.19)	0.63 (0.22)
CDR-SB	1.93 (0.96)	1.98 (1.35)	2.35 (1.62)

Data are shown as mean (SD).

† *p* < 0.01 significant difference from baseline by paired *t*-test; F, female; M, male.

Participants were stratified into younger (<70 years, n = 6) and older ( ≥ 70 years, *n* = 17) groups for exploratory analyses. Baseline demographic and clinical characteristics were comparable between the two age groups, including sex distribution, education, clinical stage, baseline MMSE score, baseline centiloid value, and vascular risk factors (all *p* > 0.05) (Table 2).

**TABLE 2:** Baseline characteristics of the study population stratified by age group.

Variable	Younger group (*n* = 6) (age < 70)	Older group (*n* = 17) (age ≥ 70)	*P*-value
Age (years)	62.0 (4.8)	75.5 (3.3)	–
Female sex, n (%)	4 (66.7)	14 (82.4)	0.56
Education (years)	11.0 (1.6)	10.7 (1.6)	0.76
Clinical stage (MCI/mild AD dementia), n (%)	6 (100)/0 (0)	14 (82) / 3 (18)	0.54
Baseline MMSE	24.8 (1.9)	25.6 (1.6)	0.42
Baseline centiloid	64.2 (15.9)	66.6 (18.0)	0.76
Hypertension, n (%)	3 (50.0)	8 (47.1)	1.00
Diabetes mellitus, n (%)	2 (33.3)	6 (35.3)	1.00
Dyslipidemia, n (%)	2 (33.3)	5 (29.4)	1.00

Continuous variables are presented as mean (SD) and categorical variables as n (%). Between-group comparisons were performed using Student’s *t*-test for continuous variables and Fisher’s exact test for categorical variables.

MMSE scores improved significantly at 6 months (*p* = 0.003, Cohen’s dz = 0.701) and showed a trend toward improvement at 12 months (*p* = 0.014, Cohen’s dz = 0.605), although this did not meet the prespecified significance threshold (*p* < 0.01) applied to account for multiple comparisons (Table 1). CDR-GS and CDR-SB scores showed slight, non-significant increases at both follow-ups.

Five patients met criteria for cognitive decline, 5 patients were classified as stable, and 13 patients showed cognitive improvement.

In the structured health interview, 19 of the 23 participants reported engaging in physical activity, social activity, or both at least twice per week.

### 3.2 Global and regional amyloid reduction on PET

**FIGURE 1:**
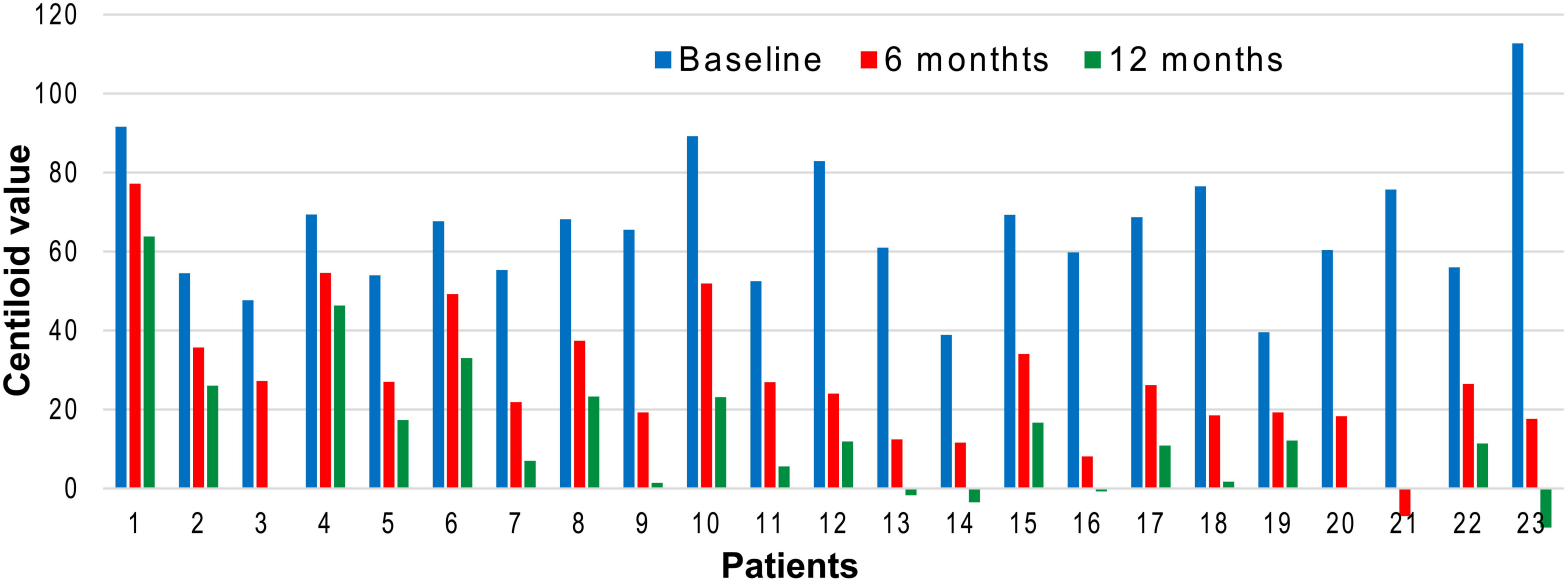
Individual centiloid trajectories during lecanemab therapy. This bar graph displays centiloid values for all 23 patients at baseline (blue), 6 months (red), and 12 months (green), arranged from left to right in order of increasing age. Amyloid burden decreased in every patient, but the magnitude of reduction varied substantially across individuals. Notably, greater centiloid reductions were generally observed in older patients (toward the right side of the graph), whereas younger patients tended to show more modest decreases, illustrating the age-dependent differences in amyloid clearance during lecanemab therapy.

**FIGURE 2:**
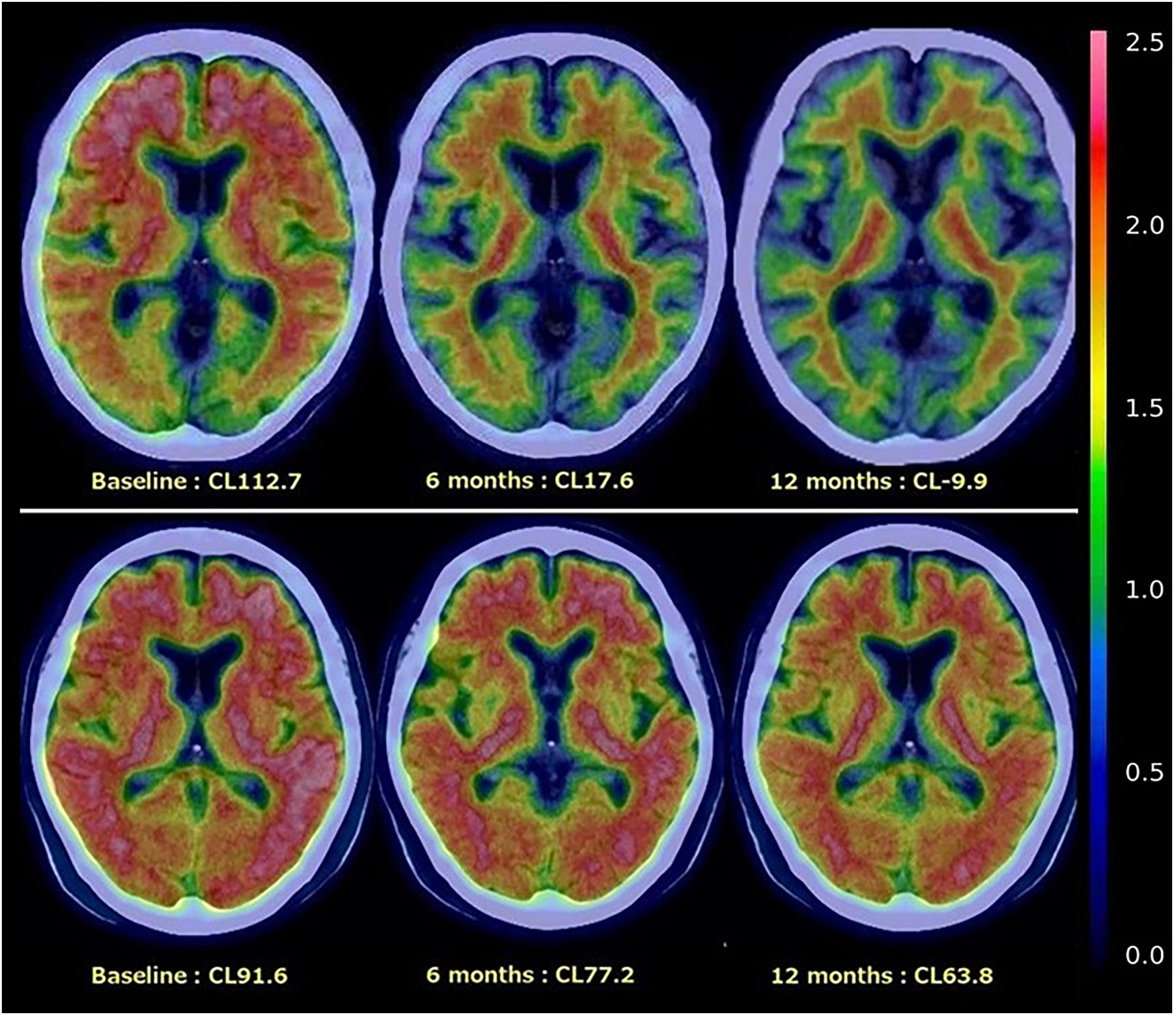
Age-related differences in amyloid reduction on serial amyloid PET. In the upper images, a woman in her 80s shows rapid and near-complete reduction in amyloid deposition over 12 months of lecanemab therapy. In contrast, the lower images depict a woman in her 50s, in whom amyloid reduction proceeds more slowly despite the same treatment protocol. The same color scale (lookup table, LUT) was applied across all time points to ensure direct comparability of PET signal intensity. CL, centiloid value.

Amyloid PET showed decreased Aβ burden in all participants after 6 months of treatment, although individual variability was considerable (Figures 1, 2).

After 12 months of treatment, 10 patients reached centiloid values below 11, and 5 patients reached values below zero. Notably, one of these patients had already become negative at the 6-month assessment (Figure 2).

Mean centiloid reduction was 38.2 at 6 months and 51.9 at 12 months (Table 1).

Regional analyses showed that the precuneus exhibited the highest baseline amyloid burden and had significantly higher SUVR compared with all other regions except the basal ganglia (Table 3).

**TABLE 3:** Regional SUVR changes and global centiloid reduction during treatment.

		Baseline	Baseline to 6 months	Baseline to 12 months
Frontal lobe	R	1.292 (0.118)	−0.243 (0.108)	−0.309 (0.137)
	L	1.275 (0.134)	−0.229 (0.136)	−0.337 (0.104)
Parietal lobe	R	1.311 (0.124)	−0.238 (0.116)	−0.320 (0.143)
	L	1.293 (0.156)	−0.222 (0.146)	−0.303 (0.168)
Temporal lobe	R	1.447 (0.109)	−0.222 (0.103)	−0.293 (0.125)
	L	1.426 (0.154)	−0.218 (0.135)	−0.300 (0.162)
Occipital lobe	R	1.307 (0.155)	−0.090 (0.069) ^†^	−0.115 (0.088) ^†^
	L	1.315 (0.139)	−0.087 (0.062) ^†^	−0.123 (0.079) ^†^
PCC	R	1.463 (0.168)	−0.230 (0.107)	−0.298 (0.120)
	L	1.427 (0.165)	−0.236 (0.118)	−0.319 (0.137)
Precuneus	R	1.567 (0.153)	−0.291 (0.135)	−0.395 (0.156)
	L	1.577 (0.160)	−0.300 (0.162)	−0.408 (0.181)
Basal ganglia	R	1.512 (0.129)	−0.225 (0.086)	−0.303 (0.117)
	L	1.521 (0.141)	−0.262 (0.108)	−0.336 (0.138)
Centiloid value	–	66.0 (17.1)	−38.2 (20.8)	−51.9 (22.7)

Data are shown as mean (SD). Each SUVR was calculated with the cerebellar cortex as the reference region. A repeated-measures ANOVA revealed significant regional differences in baseline SUVR (*p* < 0.01). The precuneus showed the highest SUVR, and *post-hoc* pairwise comparisons with Bonferroni correction confirmed that it was significantly higher than all other regions except the basal ganglia (*p* < 0.01). A repeated-measures ANOVA also demonstrated significant regional differences in amyloid reduction (*p* < 0.01).

† *Post-hoc* pairwise comparisons indicated that the occipital lobe exhibited a significantly smaller reduction than all other regions (all *p* < 0.01).

**FIGURE 3:**
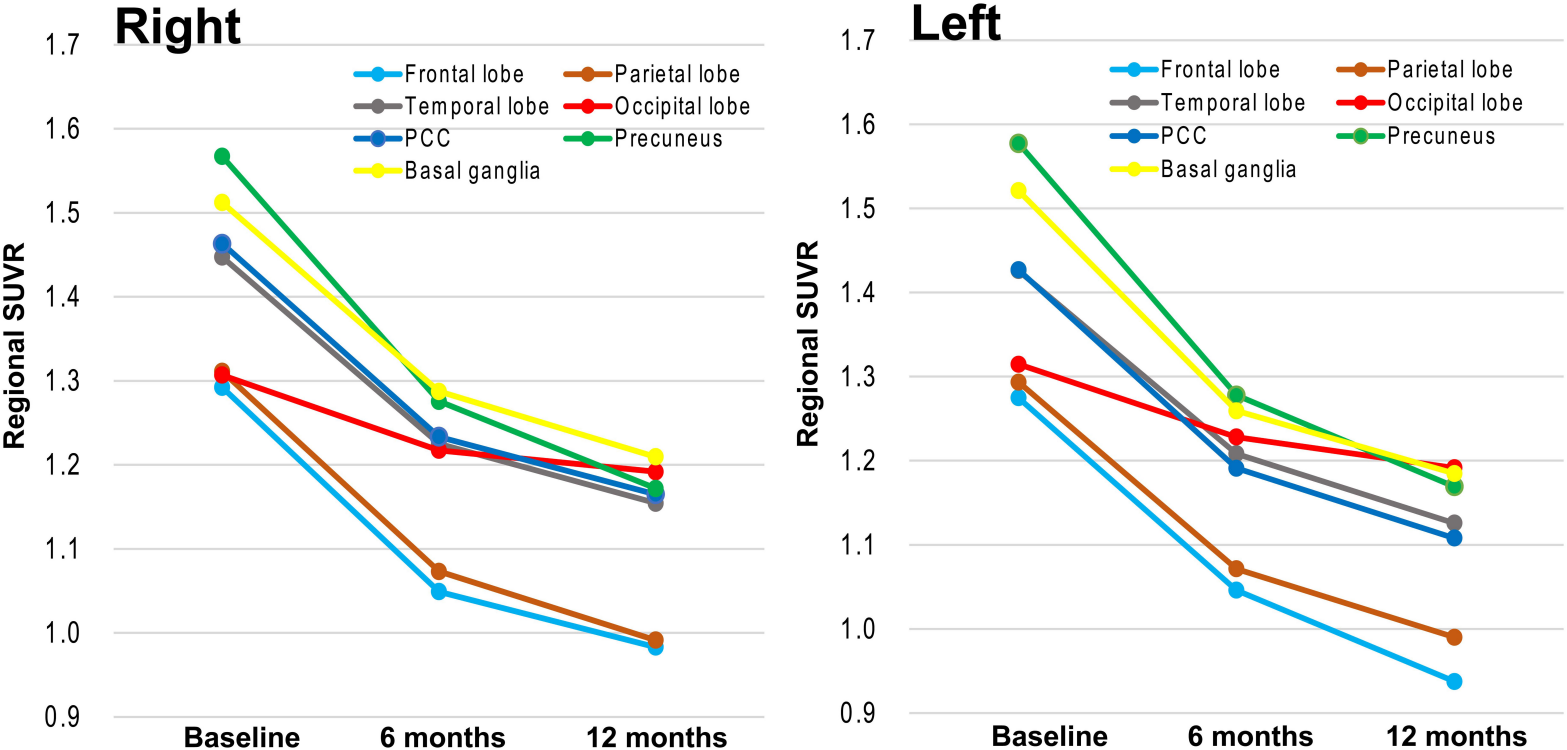
Longitudinal regional SUVR reductions during lecanemab therapy. Line plots illustrate changes in regional SUVR across baseline, 6 months, and 12 months for the **right** (left panel) and **left** (right panel) hemispheres. Although all cortical regions show progressive reductions over time, the occipital lobe (red line) exhibits the smallest decline, indicating comparatively slower amyloid clearance. In contrast, regions such as the precuneus, posterior cingulate cortex, and frontal and parietal association cortices demonstrate more pronounced SUVR reductions. These findings highlight regional variability in plaque removal kinetics, with the occipital cortex showing relative resistance to amyloid reduction. Variability of measurements is shown in Table 3 (standard deviation values).

The occipital lobe showed the smallest amyloid reduction, with significantly slower plaque removal kinetics than other regions such as the frontal, parietal, and temporal lobes, posterior cingulate cortex (PCC), precuneus, and basal ganglia (Figure 3 and Table 3). No significant interregional differences were observed apart from the occipital lobe.

### 3.3 Determinants of amyloid reduction

**FIGURE 4:**
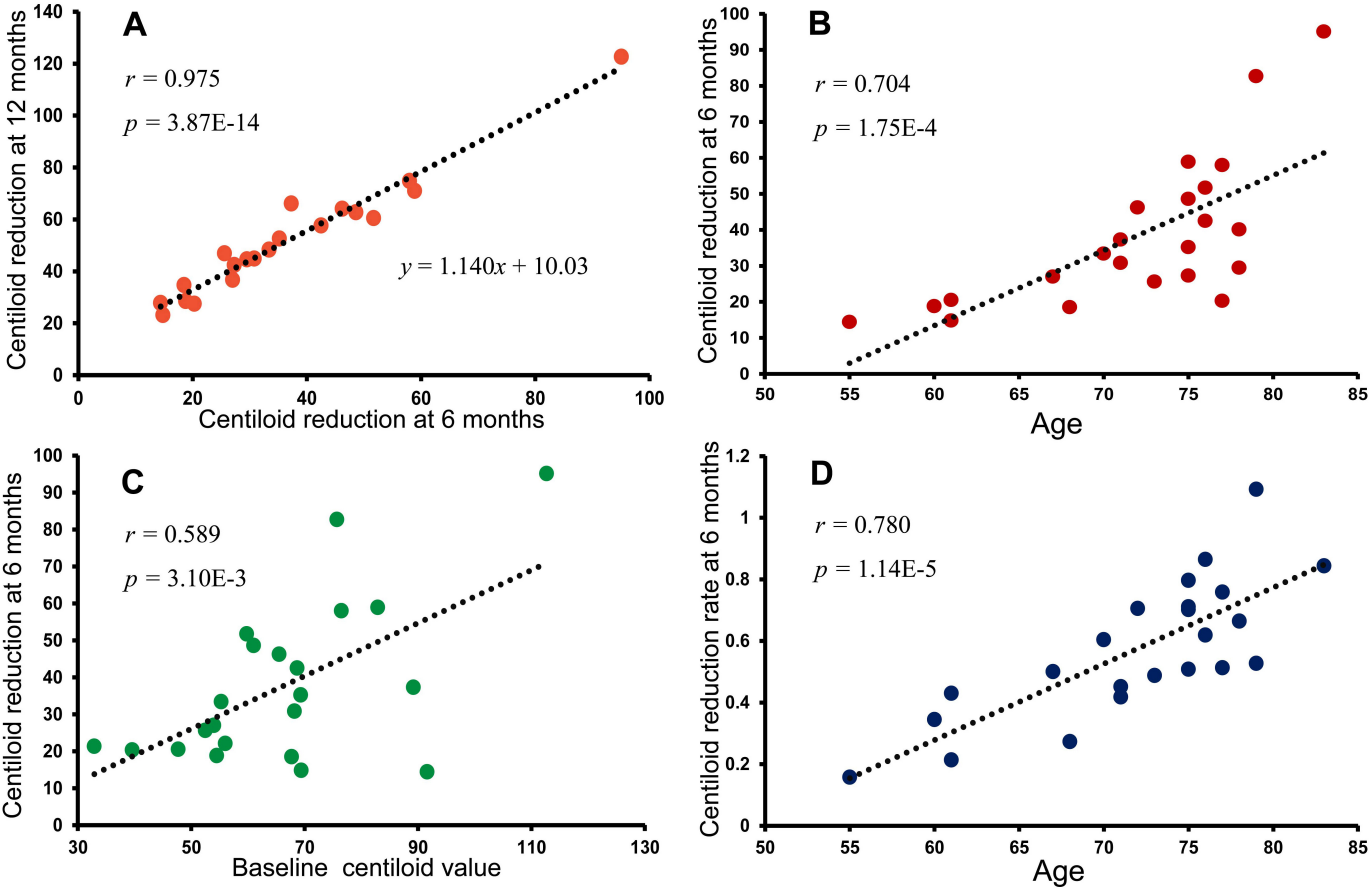
Determinants of amyloid clearance kinetics during lecanemab therapy. **(A)** Amyloid reduction at 6 months was strongly correlated with reduction at 12 months, indicating that early serial PET captures subsequent amyloid clearance trajectories. **(B)** The magnitude of amyloid reduction at 6 months was associated with age. **(C)** Baseline amyloid burden was also correlated with early reduction. **(D)** After adjustment for baseline amyloid burden, the reduction rate remained more strongly associated with age, suggesting possible age-related differences in the observable kinetics of amyloid plaque reduction.

Centiloid reduction at 6 months was strongly correlated with that at 12 months, and a linear regression model showed that 12-month amyloid reduction could be predicted from the 6-month value (Figure 4A).

The 6-month centiloid reduction was also significantly associated with age (Figure 4B) and baseline centiloid value (Figure 4C).

After adjusting for baseline centiloid, the reduction rate remained strongly associated with age (Figure 4D), with younger participants exhibiting smaller decreases, whereas older participants tended to show larger reductions.

No significant correlations were found between cognitive change and baseline or reduced centiloid values.

### 3.4 Cognitive trajectories and baseline cerebral perfusion

Five patients were classified as having cognitive decline at 6 months (Table 4). In this group, MMSE scores decreased significantly from 24.8 (0.8) to 23.2 (0.8) (paired *t*-test, *p* = 0.00284; Cohen’s *d* = 2.92), and CDR-SB scores increased from 2.40 (1.08) to 4.10 (0.96) (*p* = 0.00262; Cohen’s *d* = 2.98).

**TABLE 4:** Baseline and 6-month outcomes by cognitive trajectory group.

Group	Decline		Stable or improved	
	Baseline	6 months	Baseline	6 months
Participants (n)	5		18	
Age (y)	72.8 (7.8)		71.6 (6.7)	
Gender (M/F)	1/4		4/14	
MMSE	24.8 (0.8)	23.2 (0.8)^†^	26.0 (1.4)	28.4 (1.7)^†^
CDR-SB	2.40 (1.08)	4.10 (0.96)^†^	1.69 (0.77)	1.39 (0.86)
Centiloid value	65.0 (8.7)	25.5 (17.6)	66.2 (19.0)	28.4 (18.3)
Centiloid reduction	–	39.5 (18.4)	–	37.9 (22.0)

Data are presented as mean (SD).

**^†^** *p* < 0.01 for within-group comparisons versus baseline (paired *t*-test). The stable or improved group consisted of 5 stable and 13 improved patients. Between-group differences at baseline were not significant for age, MMSE, CDR-SB, or centiloid values. The magnitude of centiloid reduction over the first 6 months also did not differ significantly between groups, indicating that cognitive decline could not be attributed to insufficient amyloid clearance.

Baseline age and global amyloid burden were comparable between the cognitive-decline and stable/improved groups. Likewise, neither baseline centiloid values nor the magnitude of centiloid reduction differed between groups: the decline group showed a mean baseline centiloid of 65.0 (8.7) and a 6-month reduction of 39.5 (18.4), compared with 66.2 (19.0) and 37.9 (22.0), respectively, in the stable/improved group. These findings suggest that insufficient amyloid clearance was unlikely to explain cognitive deterioration.

**FIGURE 5:**
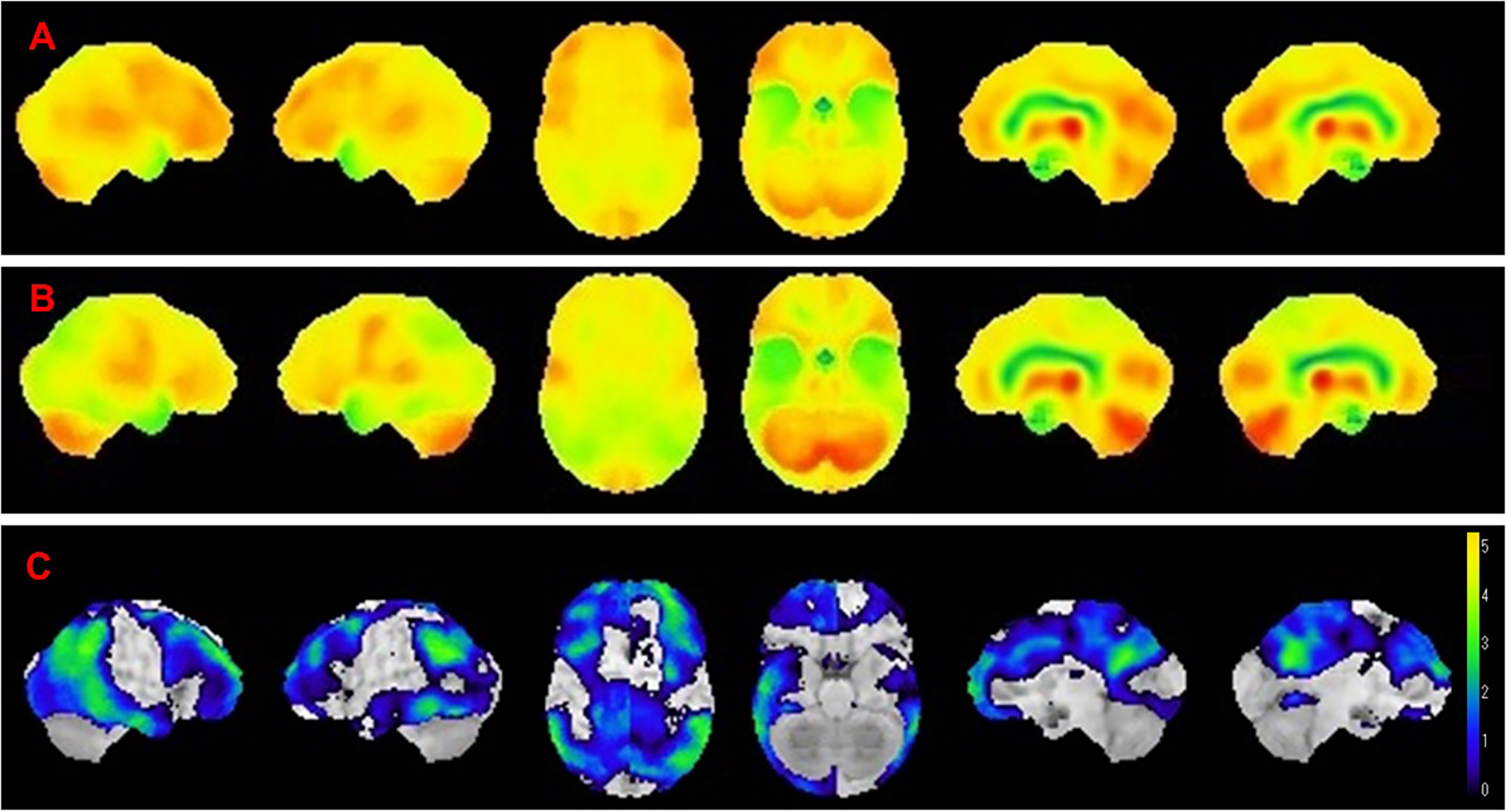
Baseline perfusion patterns identifying patients at risk for cognitive decline. Baseline cerebral perfusion patterns associated with cognitive trajectory. **(A)** The averaged baseline perfusion SPECT images of patients whose cognition improved or remained stable during lecanemab therapy, demonstrating minimal hypoperfusion. **(B)** The corresponding images in patients who experienced cognitive decline. **(C)** Z-score maps illustrating regions of significantly reduced perfusion in the decline group relative to the stable/improved group. Marked hypoperfusion is observed in the parietal and temporal association cortices, with additional frontal involvement, indicating substantial baseline neurodegenerative burden in patients who later exhibited cognitive deterioration.

In contrast, baseline cerebral perfusion SPECT revealed marked hypoperfusion in the decline group, extending from the parietal association cortex to the temporal association cortex, with additional frontal involvement. Patients whose cognition remained stable or improved showed minimal baseline hypoperfusion (Figure 5).

### 3.5 Safety

Mild, asymptomatic ARIA was observed in one patient at 6 months.

As infusion reactions, two patients developed low-grade fever in the 37 °C range that resolved overnight, and two patients experienced rash on the lower legs that disappeared within 2 days.

No other adverse events were observed.

## 4 Discussion

### 4.1 Age-dependent variability in amyloid reduction

In the present real-world cohort, lecanemab reduced insoluble amyloid deposition in all treated patients; however, the magnitude of reduction varied considerably among individuals. Notably, younger patients (<70 years) tended to exhibit relatively smaller reductions in amyloid burden on serial amyloid PET compared with older patients.

Large randomized clinical trials of lecanemab have consistently demonstrated robust reductions in amyloid burden across treated participants. In the phase 3 CLARITY-AD trial, amyloid reduction was observed broadly across demographic subgroups, including different age strata (Van Dyck et al., 2023). However, detailed analyses specifically focusing on age-related differences in the kinetics of amyloid plaque removal were not a primary focus of those trials. As a result, the potential influence of age on the dynamics of PET-detectable amyloid clearance remains incompletely characterized.

Previous longitudinal imaging studies have suggested that amyloid accumulation and turnover dynamics may vary across the lifespan. For example, longitudinal PET studies have demonstrated age-related variability in amyloid deposition and plaque progression in cognitively normal and prodromal Alzheimer’s disease populations (Jack et al., 2013; Villemagne et al., 2013). In addition, recent work has suggested that biological age acceleration may modulate regional neurodegeneration patterns in Alzheimer’s disease (Sreepada et al., 2025). These findings indicate that biological processes governing amyloid metabolism and neurodegeneration may differ across age groups. In this context, the present real-world observations raise the possibility that age-related biological variability may influence the magnitude of PET-detectable amyloid reduction during anti-amyloid therapy.

Several biological mechanisms may potentially contribute to this age-related variability. Although direct evidence remains limited, possible explanations include differences in blood–brain barrier transport, antibody penetration into the central nervous system, or age-related variation in amyloid turnover kinetics. Younger individuals may also exhibit relatively higher Aβ production rates or a different dynamic equilibrium between soluble protofibrils and insoluble plaques, which could influence the apparent rate of PET-detectable plaque reduction during therapy.

Importantly, the present findings should not be interpreted as evidence of reduced therapeutic efficacy in younger patients. Rather, the observed variability may reflect differences in the biological dynamics of amyloid metabolism across age groups.

In the present cohort, correlation analyses also suggested an association between age and the magnitude of amyloid reduction during treatment. Although baseline amyloid burden was related to early reduction magnitude, age remained associated with the rate of amyloid reduction after accounting for baseline centiloid values. Taken together, these findings raise the possibility that age may influence the observable kinetics of amyloid plaque removal during anti-amyloid therapy. However, given the relatively small sample size of this study, these observations should be interpreted cautiously and require confirmation in larger prospective cohorts. In particular, age-related differences observed in this cohort should be interpreted as exploratory signals rather than definitive biological effects.

### 4.2 Predictive value of early amyloid reduction

Serial amyloid PET analyses showed that amyloid reduction at 6 months was strongly associated with that at 12 months, suggesting that early longitudinal imaging may capture important features of subsequent amyloid dynamics during lecanemab therapy (Figure 4A). The regression analysis demonstrated a close relationship between early and later PET changes, indicating that early PET measurements may provide useful information regarding longer-term amyloid trajectories rather than reflecting transient effects.

The regression coefficient approaching unity suggests that PET-detectable amyloid reduction may slow in the latter phase of treatment despite continued dosing, implying that a substantial proportion of measurable amyloid change may occur during the early treatment period and can be captured by interim PET assessment. Because early centiloid reduction was also associated with baseline amyloid burden (Figure 4C), the magnitude of reduction achieved within the first 6 months appeared to influence subsequent PET values at 12 months.

Taken together, these findings suggest that early serial amyloid PET may serve as a useful tool for treatment monitoring and disease tracking in real-world clinical practice, enabling individualized longitudinal assessment without implying differences in intrinsic therapeutic efficacy.

### 4.3 Protofibril targeting and plaque clearance dynamics

After 12 months of lecanemab therapy, eight patients achieved amyloid levels below 11 centiloids, consistent with near-complete plaque clearance (La Joie et al., 2019). Neuropathological findings from a phase 2 trial similarly showed minimal diffuse and neuritic plaques in a lecanemab-treated individual (Honig et al., 2022), supporting the concept that protofibril neutralization precedes fibrillar amyloid removal. Although lecanemab binds soluble protofibrils with much higher affinity than plaque-associated Aβ, the degree of plaque reduction observed in our cohort was substantial, except in younger individuals.

This may reflect recognition of protofibril-like epitopes within plaques (Swanson et al., 2021) and disruption of the equilibrium among Aβ species, whereby protofibril depletion drives secondary plaque dissolution. While amyloid PET primarily reflects fibrillar Aβ, mAb158—the murine precursor of lecanemab—reduced protofibrils before measurable plaque clearance in mouse models (Rizoska et al., 2024), suggesting that PET-detectable plaque reduction in humans follows earlier depletion of soluble protofibrils. These findings underscore the therapeutic relevance of targeting early Aβ species in AD (Hampel et al., 2021).

### 4.4 Regional differences in amyloid clearance

Amyloid reduction in the occipital lobe was significantly smaller than in other cortical regions, whereas no significant differences were observed across remaining areas (Figure 3). The relatively preserved occipital amyloid burden may reflect region-specific plaque composition or the presence of vascular amyloid due to cerebral amyloid angiopathy, both of which could impede antibody accessibility and slow clearance (Attems and Jellinger, 2007; Attems and Jellinger, 2005). Although plausible, this interpretation remains speculative and requires validation through dedicated imaging and neuropathological studies.

### 4.5 Cognitive decline despite adequate amyloid clearance

Cognitive decline despite substantial amyloid reduction has been reported in studies of anti-amyloid therapies and longitudinal imaging cohorts, highlighting heterogeneity in clinical responses to treatment (Van Dyck et al., 2023; Villemagne et al., 2013). Five patients exhibited cognitive decline despite substantial amyloid reduction during lecanemab therapy. Their baseline centiloid values and the magnitude of amyloid reduction over the first 6 months were comparable to those observed in patients with stable or improved cognition, indicating that insufficient amyloid clearance was unlikely to account for subsequent cognitive deterioration.

In contrast, baseline cerebral perfusion SPECT revealed marked hypoperfusion involving the parietal and temporal association cortices, with additional frontal involvement—regions commonly implicated in tau-associated neurodegeneration. Given that tau accumulation has been shown to correlate with reduced glucose metabolism (Hanseeuw et al., 2017), and that cerebral blood flow reflects regional metabolic activity (Fox and Raichle, 1988), these perfusion abnormalities are consistent with a substantial downstream neurodegenerative burden present at treatment initiation.

Although perfusion SPECT does not directly quantify tau deposition, it may serve as a practical interim surrogate for tau-related neurodegeneration in real-world clinical settings where tau PET is not readily available. From a clinical perspective, baseline perfusion imaging may therefore enable meaningful risk stratification by identifying patients in whom downstream neurodegeneration predominates despite adequate amyloid removal. Early identification of such patients could support closer monitoring, timely implementation of supportive interventions, and prioritization for emerging tau-targeted or combination therapeutic strategies. In addition, because cognitive assessments were conducted as part of routine clinical care, raters were not formally blinded to PET results, which could introduce potential bias in clinical outcome evaluation.

### 4.6 Safety considerations

In this real-world cohort, lecanemab showed a favorable safety profile, with only one case of asymptomatic ARIA. In the CLARITY-AD Asian subgroup (including Japanese participants), ARIA rates were lower than in the global cohort (ARIA-E: 6.2% vs. 12.6%; ARIA-H: 14.4% vs. 17.3%) (Chen et al., 2025), suggesting potentially lower ARIA risk in Asian populations. Nevertheless, ongoing MRI surveillance and structured risk management remain essential.

## 5 Limitation

This study has several limitations. First, the cohort size was modest and drawn from a single specialized center. Although this may constrain generalizability, the uniform directionality of amyloid reduction and the large effect sizes enhance confidence in the biological conclusions. These findings should therefore be interpreted as hypothesis-generating and warrant confirmation in larger multicenter studies investigating age-related heterogeneity in amyloid clearance during anti-amyloid therapy.

Second, the 12-month follow-up period, while sufficient to establish the predictive value of early PET and to characterize amyloid kinetics, does not capture longer-term cognitive or neurodegenerative trajectories. Extended follow-up will be necessary to determine whether early biological effects translate into durable clinical benefit.

Third, cognitive assessments (MMSE, CDR-SB) may be influenced by practice effects and mild baseline disease severity. In particular, MMSE is a relatively coarse measure of cognitive performance and may have limited sensitivity for detecting subtle cognitive changes. Additionally, real-world care at our center included structured lifestyle guidance encouraging physical and social activity, which may have contributed to favorable cognitive outcomes and complicates direct comparison with controlled trials.

Fourth, although perfusion SPECT was used to approximate neurodegenerative burden, tau PET would provide a more direct assessment of tau pathology. Tau PET was not feasible in this real-world clinical setting, and tau biomarkers were not routinely available in this cohort. Therefore, the biological diagnosis relied primarily on amyloid PET together with clinical evaluation and structural MRI findings. Future studies incorporating tau biomarkers will be important to further refine biological disease characterization.

Finally, although age was strongly associated with amyloid reduction, the observational design precludes causal inference. Age-related differences likely reflect variability in amyloid clearance kinetics or pharmacokinetic factors rather than differences in intrinsic treatment efficacy. Larger studies integrating multimodal biomarkers will be required to clarify these mechanisms and guide age-adapted therapeutic strategies.

## 6 Conclusion

In this real-world cohort, lecanemab treatment resulted in substantial reductions in amyloid burden detectable by serial amyloid PET. The magnitude of amyloid reduction appeared to vary with age, suggesting possible age-related differences in amyloid clearance kinetics during therapy. In contrast, short-term cognitive trajectories were more closely associated with baseline cerebral perfusion patterns than with the degree of amyloid reduction. These findings highlight the potential value of multimodal imaging in understanding biological heterogeneity and optimizing patient stratification during anti-amyloid therapy.

## Data Availability

The raw data supporting the conclusions of this article will be made available by the authors, without undue reservation.
